# Modelling the Dynamics of Post-Vaccination Immunity Rate in a Population of Sahelian Sheep after a Vaccination Campaign against Peste des Petits Ruminants Virus

**DOI:** 10.1371/journal.pone.0161769

**Published:** 2016-09-07

**Authors:** Pachka Hammami, Renaud Lancelot, Matthieu Lesnoff

**Affiliations:** 1 UMR Contrôle des Maladies Animales Exotiques et Emergentes (Cmaee), Centre de coopération internationale en recherche agronomique pour le développement (Cirad), Campus international de Baillarguet, 34398 Montpellier, France; 2 UMR Cmaee 1309, Institut national de la recherche agronomique (Inra), Campus international de Baillarguet, 34398 Montpellier, France; 3 UMR Systèmes d’élevage méditerranéens et tropicaux (Selmet), Cirad, Campus international de Baillarguet, 34398 Montpellier, France; 4 UMR Selmet, Inra, Campus international de Baillarguet, 34398 Montpellier, France; 5 UMR Selmet, Montpellier Supagro, Campus international de Baillarguet, 34398 Montpellier, France; University of Minnesota, UNITED STATES

## Abstract

**Background:**

Peste des petits ruminants (PPR) is an acute infectious viral disease affecting domestic small ruminants (sheep and goats) and some wild ruminant species in Africa, the Middle East and Asia. A global PPR control strategy based on mass vaccination—in regions where PPR is endemic—was recently designed and launched by international organizations. Sahelian Africa is one of the most challenging endemic regions for PPR control. Indeed, strong seasonal and annual variations in mating, mortality and offtake rates result in a complex population dynamics which might in turn alter the population post-vaccination immunity rate (*PIR*), and thus be important to consider for the implementation of vaccination campaigns.

**Methods:**

In a context of preventive vaccination in epidemiological units without PPR virus transmission, we developed a predictive, dynamic model based on a seasonal matrix population model to simulate *PIR* dynamics. This model was mostly calibrated with demographic and epidemiological parameters estimated from a long-term follow-up survey of small ruminant herds. We used it to simulate the *PIR* dynamics following a single PPR vaccination campaign in a Sahelian sheep population, and to assess the effects of (i) changes in offtake rate related to the Tabaski (a Muslim feast following the lunar calendar), and (ii) the date of implementation of the vaccination campaigns.

**Results:**

The persistence of *PIR* was not influenced by the Tabaski date. Decreasing the vaccination coverage from 100 to 80% had limited effects on *PIR*. However, lower vaccination coverage did not provide sufficient immunity rates (*PIR* < 70%). As a trade-off between model predictions and other considerations like animal physiological status, and suitability for livestock farmers, we would suggest to implement vaccination campaigns in September-October. This model is a first step towards better decision support for animal health authorities. It might be adapted to other species, livestock farming systems or diseases.

## Introduction

### Peste des petits ruminants

Peste des petits ruminants (PPR) is an acute infectious viral disease affecting domestic small ruminants (sheep and goats), and some wild ruminant species [1]. It is caused by a Morbillivirus, the PPR virus (PPRV). Widespread in Africa, the Middle East and Asia, it causes heavy economic losses, mostly in smallholder, low-input farming systems [2–4].

Following the recent eradication of rinderpest (a cattle disease caused by another Morbillivirus) in 2011 [5], PPR eradication is now a top priority for improving animal health and farmers livelihood, making it a Global Public Good [6]. A global strategy for the progressive control and eradication of PPR was launched in 2015 by the World Organization for Animal health (OIE) and the Food and Agriculture Organization of the United Nations (FAO) [7]. In areas where PPR is endemic, like in the Sahelian region of Africa, vaccination is the primary tool for PPR control.

In Sahelian Africa, the most commonly used PPR vaccine is the attenuated Nigeria 75/1 strain of PPRV which provides, after a single injection, a life-long immunity against PPRV [8–10]. Moreover, vaccinated ewes and nanny goats, as well as those recovering from a natural PPRV infection, provide their offspring with maternal (colostral) antibodies. These kids and lambs are thus protected against PPRV during the first months of their life [11–13]. Because of this passive immunity, as well as the immaturity of their immunized system [14], only animals older than three months are vaccinated, thus defining the target population for PPR vaccination [7].

The recommended strategy in Sahelian Africa is to vaccinate the whole target population during one or two successive years (a single vaccination round each year), followed by the vaccination of the sole offspring (animals < 1 year old) during one or two successive years [7]. Each vaccination round must be implemented over a short period of time, preferably at the beginning of the dry season (November-December) and before the birth peak [7] when grazing is abundant and animals in good health.

### Post-vaccination immunity rate

After such a pulse vaccination campaign, the population immunity rate (*PIR*), i.e. the proportion of immunized individuals gradually decreases according to the population demographic turnover: births and other unvaccinated entries on the one hand, and deaths and offtake (slaughtering and sales) on the other hand. Knowledge on the post-vaccination immunity decay between two successive vaccination campaigns is important to assess the efficiency of vaccination programs, and to provide veterinary services with recommendations on vaccination strategies. In the absence of PPRV transmission, the post-vaccination *PIR* dynamics only depends on the population turnover. Indeed, at a given time after the vaccination, immunized animals may have been affected by demographic events, such as death and offtake. Therefore, their number is decreasing with time. In the meantime, the number of unvaccinated animals (births, purchases and loans from unvaccinated and PPR-free areas…) is growing. Moreover, the persistence of maternal antibodies is short: a few months after birth [13, 15, 16]. Consequently, the *PIR* is decreasing in the vaccinated population. How fast? To answer this complex question in a context of preventive vaccination against avian influenza in free-range domestic poultry, Lesnoff *et al*. [17] used a discrete-time population matrix model. As a matter of fact, such models are commonly used for simulating the dynamics of age-structured populations [18–20], and represent epidemiological processes [21, 22]. We chose this approach for this study.

In extensive sheep farming systems, the availability of forage resources is closely related to the nutritional and physiological status of animals, and therefore the reproductive performances [23]. More specifically, in the Sahelian region, sheep physiological needs are only met during the rainy season, from July to September. Therefore, mating is mostly limited to this time period resulting in a single birth peak between December and February [24].

Moreover, offtake rates are also highly seasonal with a large increase during the Tabaski festival (Eid al-Adha), a religious celebration during which young rams are sacrificed in most families [24–27]. According to the Gregorian calendar, the offtake peak moves backward by nearly two weeks each year because the Tabaski date is based on the lunar (shorter) calendar. These seasonal patterns in demographic parameters may affect the small ruminant population dynamics, and consequently the *PIR* dynamics.

### Post-vaccination immunity threshold

The basic reproduction number *R*_0_ is the expected number of new infections following the introduction of a single infectious individual in a population of fully susceptible hosts [28]. Assuming an homogeneous and randomly-mixed small ruminant population, as well as a long-lasting immunity in vaccinated animals [9], the fraction (*f*) of immunized individuals required to stop PPRV-transmission can be estimated by *f* > 1 − 1/*R*_0_ [28, 29]. Epidemiological studies implemented in smallholder farming systems provided empirical *R*_0_ estimates ranging from 4.0 to 6.9 [30–32], with corresponding *f* ranging from 75% to 86%. On the other hand, Moroccan veterinary services successfully controlled PPR with three successive annual, nation-wide mass vaccination campaigns following PPR emergence in 2008. No PPR outbreak was detected after the end of the first vaccination campaign (more than 20 million sheep and goats vaccinated in October-November 2008) which covered 85% of the national stock. In March 2009, the estimated post-vaccination seroprevalence rate of antibodies against PPRV was 69% (*n* = 5,158) in small ruminants [33]. Consequently, it was agreed among OIE and FAO experts in charge of designing the recommended PPR control strategy, that PPR mass vaccination campaigns should target a post-vaccination *PIR* of at least 70%. This threshold was chosen to assess the success of PPR vaccination campaigns through post-vaccination monitoring activities at the level of epidemiological units [7].

In the context of PPR vaccination campaigns in Sahelian small-ruminant farming systems, the epidemiological unit can be arbitrarily defined as an area and its corresponding small-ruminant population covered by a vaccination team during a vaccination session (generally from one to three days). The epidemiological unit has a variable size according to the local conditions. However, in the commonest situations, it ranges from a single village or settlement, to a municipality (encompassing several villages), representing from several hundreds to several thousands of sheep and goats. In such an epidemiological unit, the small ruminant population can be considered as homogeneous and randomly mixed with respect to the vaccination probability, as well as with the risk of PPRV transmission.

### Study goals

The goal was to assess the persistence of *PIR* in sheep after a single mass-vaccination campaign, according to different preventive vaccination scenarios, in an epidemiological unit without PPRV transmission (i.e. PPR vaccination and colostral antibodies were the only sources of immunized sheep). Specific objectives were to assess the effects of (i) seasonality of demographic rates and (ii) vaccination date on the *PIR* dynamics.

## Materials and Methods

### Study area and sheep demographic data

**Fig 1:**
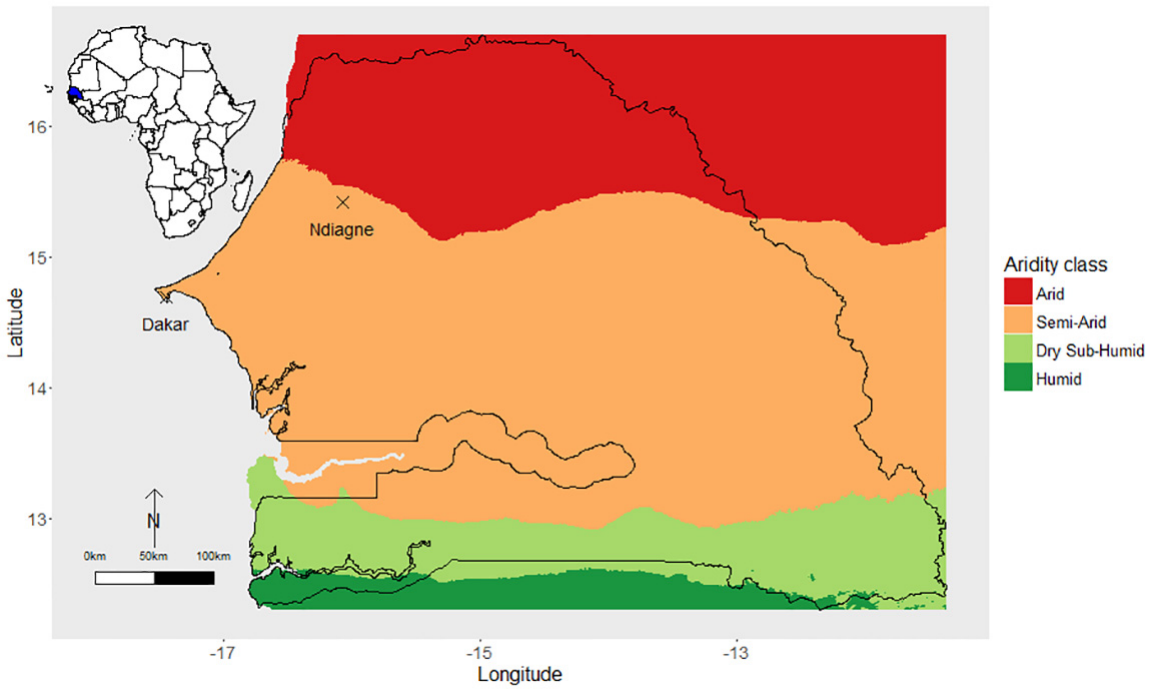
Global Aridity Index in Senegal (West Africa). Data sources: Zomer *et al*., 2006 [38] and Trabucco *et al*., 2009 [39]; spatial resolution: 10 arc minutes. The location of Senegal is shown in blue on the map of Africa in the top-left corner.

Sheep demographic data were collected during a joint research program implemented by the Senegalese Institute of Agricultural Researches (ISRA) and the French Agricultural Research Centre for International Development (CIRAD). A demographic longitudinal survey was implemented from 1983 to 1999 in more than 200 small ruminants herds belonging to 15 villages located in the Ndiagne municipality, located in northern Senegal (Fig 1) [24–26]. Climate is characterized by a short rainy season (July to September) and a long dry season. The annual rainfall ranges from 250 to 500 mm, with a rainfall peak generally observed in August [34].

Sheep were mostly from the Sahelian phenotype—so-called Peul-Peul breed: mid-size, short-hair sheep with black and white robe. They were reared in a low-input smallholder farming system relying on the utilization of natural grasslands. Herds were fortnightly visited by professional surveyors. Individual demographic events (parturition, mortality, sales, etc.) were recorded with the help of the farmer and people in charge of animal cares (in general: farmer’s wife and children). Data were stored in a purposively-designed relational database [35], and pre-processed with specific routines described in Lesnoff *et al*., 2014 [36]. The time period considered for the present study ranged from 1989 to 1995 during which all sheep were vaccinated each year against PPR with a heterologous vaccine against rinderpest virus [37]. No PPR outbreak was recorded in the vaccinated sheep flocks during the study period. Therefore, we assumed neither PPR-specific mortality, nor other PPR-related demographic effect, affected the sheep demographic rates. The observed demographic data are provided as MS Excel file in S1 Table.

### Demographic matrix model

The sheep population dynamics and age structure were simulated over one year with a seasonal discrete-time population matrix model [17, 20, 40, 41] using a one-month time step, and splitting the population by sex and one-month age classes. Shorter time steps can be used, e.g. one- or two-week time step [24], but the one-month time step allowed more parsimony in the model (smaller number of parameters) and was well adapted for the study objectives, with monthly *PIR* estimates as the output.

For a given month *j*, and with *t* representing the start of the month, the one-month dynamics was given by:

$$\begin{array}{c} x\left(t+1\;\text{month}\right)=B_{j}\times x\left(t\right) \end{array}$$

where

— *x*(*t*) was the population-state vector at time *t*. Its components are the animal numbers in the population by sex-and-age class from which the age structure can be estimated.
— The one-month projection matrix *B*_*j*_ contained the one-month demographic rates (reproduction, mortality and offtake) by sex-and-age class.

With *t* representing the start of the year, the one-year dynamics was given by:

x(t+1year)=A×x(t)

where *A* = *B*_12_ × *B*_11_ × … × *B*_1_ was the one-year projection matrix. The matrices *B*_*j*_ were filled with demographic parameters changing with the month. However, they all had the same structure (see, S1 Matrix).

Survival and net fecundity rates *s* and *f* were estimated for each sex-and-age class from the Ndiagne sheep data set. Rate *ρ*, i.e. the probability that an offspring born alive was a female, was set to 0.5 [42–44]. The age at first parturition was set to 10 months [45]. The oldest ages for ewes and rams were set to 11 years and two years [24, 25, 27, 46, 47]. All the sheep surviving up to these ages in the model were assumed to be culled, and were thus added to the offtake.

The population dynamics was simulated according to 12 so-called Tabaski scenarios. For each scenario, the male offtake rates in the *B*_*j*_ matrices were adapted to simulate the occurrence of the Tabaski festival at a different month. A birth-flow type was assumed for the monthly reproduction pattern [20]. For a given month (*t*, *t* + 1), the dynamics equations were (after removing index *j* for simplicity):

$$\begin{array}{c} x_{f,1}\left(t+1\right)=\rho \;s_{0}\sum_{i=11}^{132}f_{f,i}\;s_{f,i}\left(t\right) \end{array}$$ (1)

$$\begin{array}{c} x_{f,2}\left(t\right)=s_{f,1}\times x_{f,1}\left(t\right) \end{array}$$ (2)

$$\begin{array}{c} x_{f,...}\left(t\right)=s_{f,...}\times x_{f,...}\left(t\right) \end{array}$$ (3)

$$\begin{array}{c} x_{f,132}\left(t\right)=s_{f,131}\times x_{f,131}\left(t\right) \end{array}$$ (4)

$$\begin{array}{c} x_{m,1}\left(t+1\right)=\left(1-\rho \right)\;s_{0}\sum_{i=11}^{132}f_{f,i}s_{f,i}\left(t\right) \end{array}$$ (5)

$$\begin{array}{c} x_{m,2}\left(t\right)=s_{m,1}\times x_{m,1}\left(t\right) \end{array}$$ (6)

$$\begin{array}{c} x_{m,...}\left(t\right)=s_{m,...}\times x_{m,...}\left(t\right) \end{array}$$ (7)

$$\begin{array}{c} x_{m,24}\left(t\right)=s_{m,23}\times x_{m,23}\left(t\right) \end{array}$$ (8)

where

— Age class *i*, for *i* ≥ 1 at time *t*, represented animals having an exact age ranging between *i* − 1 months and *i* months at this time. “Age class” index 0 represented the births between *t* and *t* + 1.
— *s*_*f*,*i*_ and *s*_*m*,*i*_, for *i* ≥ 1, were the female and male survival probabilities between *t* and *t* + 1 from age class *i* to age class *i* + 1. Rates *s*_*f*,0_ and *s*_*m*,0_ were the female and male survival probabilities from the birth to age class 1. For each sex-and-age class, the survival probability *s* was calculated by *s* = 1 − *p*_*dea*_ − *p*_*off*_ where *p*_*dea*_ was the probability of natural death and *p*_*off*_ the probability of offtake.
— *f*_*f*,*i*_ was the net fecundity rate for age class *i*, i.e. the average number of offspring born alive between *t* and *t* + 1 expected per female in age class *i*.
— *ρ* was the probability that an offspring born alive was a female.

### Calculating the post-vaccination immunity dynamics from the demographic matrix model

The *PIR* at *t*, noted *PIR*(*t*), was estimated over the discrete time-scale *t* = 0 month, one month, …, 12 months of the simulated year, where *t* = 0 corresponds to the pulse vaccination round. For a given time *t*, *PIR*(*t*) was calculated from the population vector *x*(*t*) by the ratio between the number of immunized animals in the population over the total number of animals in the population. The calculations used almost the same principle as described in the Appendix of Lesnoff *et al*. [17].

Lesnoff *et al*. [17] only considered immunity provided by vaccination. Moreover, they assumed all the animals in the target population were vaccinated during the vaccination campaign. Here, we accounted for different immunization ways, as well as variable vaccination coverage (i.e. the probability for an animal to be properly vaccinated).

1. Three levels of vaccination coverage were considered: the probability to be vaccinated (*p*) was set at *p* = 1 (full vaccination coverage), *p* = 0.8, or *p* = 0.6. Partial vaccination might be related to sheep escaping the vaccination, lack of immunized response after vaccination (sick animals, poor physiological condition, etc.), inefficient vaccine due to breaks in the cold chain, etc.
2. Assuming no PPRV transmission, no new immunization (at the exception of newborn lambs with colostral immunity) and no additional mortality due to the disease were considered after the vaccination. However, we allocated an initial immunity rate at the vaccination time (start of the simulation) to account for past PPR outbreaks, and resulting post-infection immunity in recovered animals (as well as colostral antibodies in offspring born from recovered ewes). We estimated the initial immunity rates (*π*) from a literature review (Table 1).
3. PPR colostral antibodies may be detected in the serum of offspring (born from a immunized ewe) with a competition enzyme linked immunosorbent assay (cELISA) [48] up to four months [13, 15]. According to Bodjo et al., 2006 [13], during the first month of their life, only 92% of the lambs born from immunized ewe are carrying maternal antibodies; this proportion is decreasing with age, down to 5% at four months. We used this information to estimate the number of immunized newborn in the populations as follows:
  - the total number of newborn lambs was computed using the demographic matrix model,
  - it was then multiplied by the proportion of immunized reproductive ewes,
  - this latter number was multiplied by the probability (*ν*_*i*_) to be immunized by colostrum if born from an immunized ewe. For the age classes 1, 2 and 3, *ν*_*i*_ were respectively set to 0.92, 0.84, and 0.32 [11–13, 15].

**Table 1:** Initial serological prevalence rates used in the model.

Age	Serological prevalence rate (%)	References
age ≤ 6 months	33	[49, 50]
6 < age ≤ 24 months	31	[49–52]
age > 24 months	47	[49–53]

**Fig 2:**
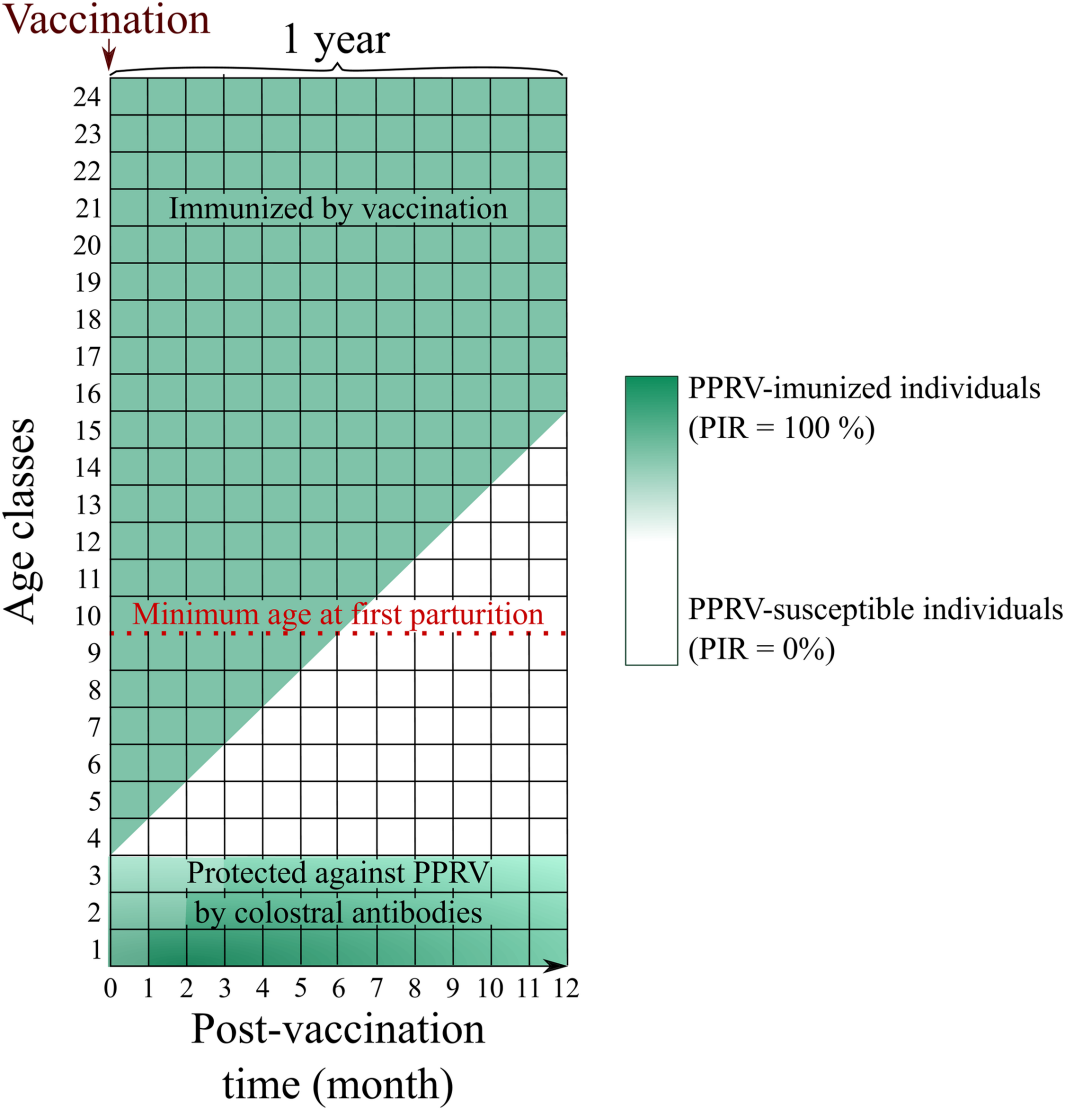
Theoretical immunity dynamics by age class (for a given sex) over 12 months after a vaccination campaign. The one-month age classes are represented by the space between two horizontal lines. The age structure of the population is represented at each time *t* by the vertical lines. Each portion of the vertical lines (between two horizontal lines) represented the animals who composed a given age class. Green and white areas represent immunized and susceptible animals. For clarity, the age has been truncated to 24 months.

For each time *t*, the number of immunized sheep in each age class *i* and sex s (with s = *f* or *m*), noted $n_{s,i}\left(t\right)$, was calculated by multiplying the total number of sheep in each age class with the corresponding probability to be immunized. This probability was differently computed for two categories of animals: (i) sheep targeted by the vaccination (i.e. in age classes *i* > 3 at *t* = 0), represented by a green triangle in Fig 2, and (ii) the others (i.e. in age classes *i* ≤ 3 at *t* = 0 and the lambs born at *t* > 0), represented by a green rectangle and a white triangle in Fig 2.

For the first category, at time *t* = 0, the probability for a sheep of age class *i* to be immunized (noted *θ*_*i*_(0)) was the sum of the probability to be immunized by a previous infection or vaccination and the probability for non-immunized sheep to be vaccinated, thus giving: *θ*_*i*_(0) = *π*_*i*_ + (1 − *π*_*i*_) × *p*. Because no new infection or additional vaccination were considered, and post-vaccination (or post-infection) immunity has a long persistence at the individual level: >> one year [10, 12, 54], this probability did not vary during the one-year simulations. Therefore:

$$\begin{array}{ccc} \theta_{i}\left(0\right) & = & \theta_{i+1}\left(1\right)=\theta_{i+2}\left(2\right)=\text{etc.} \end{array}$$

Consequently, the numbers of immunized sheep of age classe *i* and sex s at the successive times *t* were calculated as follows:

$$\begin{array}{ccc} n_{i}\left(0\right) & = & x_{s,i}\left(0\right)\times \theta_{i}\left(0\right)\\ n_{i}\left(1\right) & = & x_{s,i}\left(1\right)\times \theta_{i-1}\left(0\right)\\ & \ldots & \\ n_{i}\left(t\right) & = & x_{s,i}\left(t\right)\times \theta_{i-t}\left(0\right) \end{array}$$

where the animal numbers ($x_{s,i}\left(t\right)$) were derived from the population matrix model.

In the second category, animals were not targeted by the vaccination but were potentially immunized by the maternal antibodies during the first months of their life. The calculations of the probability to be immunized for these animals were more complex than for the first category. They jointly accounted for the probability for a newborn lamb to be born immunized at time *i* (*β*(*t*), not detailed here, it depended both on (i) the number of newborn produced by a reproductive female and the proportion of immunized reproductive females in the population at time *t* − 1), and (ii) the decay (probability *ν*) of colostral antibodies in lambs from birth to the age of three months. Consequently:

$$\begin{array}{ccc} n_{1}\left(t\right) & = & x_{s,1}\left(t\right)\times \beta \left(t\right)\times \nu_{1}\\ n_{2}\left(t\right) & = & x_{s,2}\left(t\right)\times \beta \left(t-1\right)\times \nu_{2}\\ n_{3}\left(t\right) & = & x_{s,3}\left(t\right)\times \beta \left(t-2\right)\times \nu_{3}\\ n_{i>3}\left(t\right) & = & x_{s,i>3}\left(t\right)\times \beta \left(t-i+1\right)\times 0 \end{array}$$

The *PIR*(*t*) were estimated for a total of 432 scenarios: 12 scenarios of Tabaski month (from January to December) × 12 scenarios of vaccination month (from January to December) × three scenarios of vaccination coverage efficiency (*p* = 1, *p* = 0.8, *p* = 0.6):

$$\begin{array}{c} PIR\left(t\right)=\frac{\sum_{i}n_{s,i}\left(t\right)}{\sum_{i}x_{s,i}\left(t\right)} \end{array}$$

where *t* = 0,1,…,12, with *t* = 0 representing the population immunity state at the time of the vaccination.

Four indicators were considered to summarize and compare the estimates:

— *Pvacc*: the proportion of sheep belonging to the vaccinated cohorts (older than three months) during the vaccination campaign (i.e. at *t* = 0).
— *N*_70_: the number of months for which *PIR*(*t*) was higher than or equal to 70%, over the year.
— *PIR*(12): the final population immunity rate (i.e. one year after the vaccination campaign).
— *M*_*PIR*_: the mean population immunity rate over the year: $M_{PIR}=\frac{1}{12}\sum_{t=1}^{12}PIR\left(t\right)$.

### Statistical estimation of model parameters and *PIR* simulations

Demographic rates of the population matrix model (components of matrices *B*_*j*_; *j* = 1,…,12) were estimated from the observed demographic data, using a model-averaging and multi-model inference framework to account for model selection uncertainty, i.e. related to the selection of the dependent variables in the statistical models [55]. The candidate statistical models for model selection and multi-model inference were binomial logistic or Poisson log-linear regression models depending on the demographic rate. They were fitted by the maximum likelihood [56, 57] and ranked according to the Akaike information criterion with small-sample correction (AICc), or quasi-AICc (QAICc). The latter was used if over-dispersion—with respect to the expected binomial or Poisson distribution—was observed in the data due, to herd-clustering effects [58]. The (Q)AICc differences between the “best” model and the following were used to compute the Akaike weights, forming themselves the basis for model averaging and multi-model inference.

**Fig 3:**
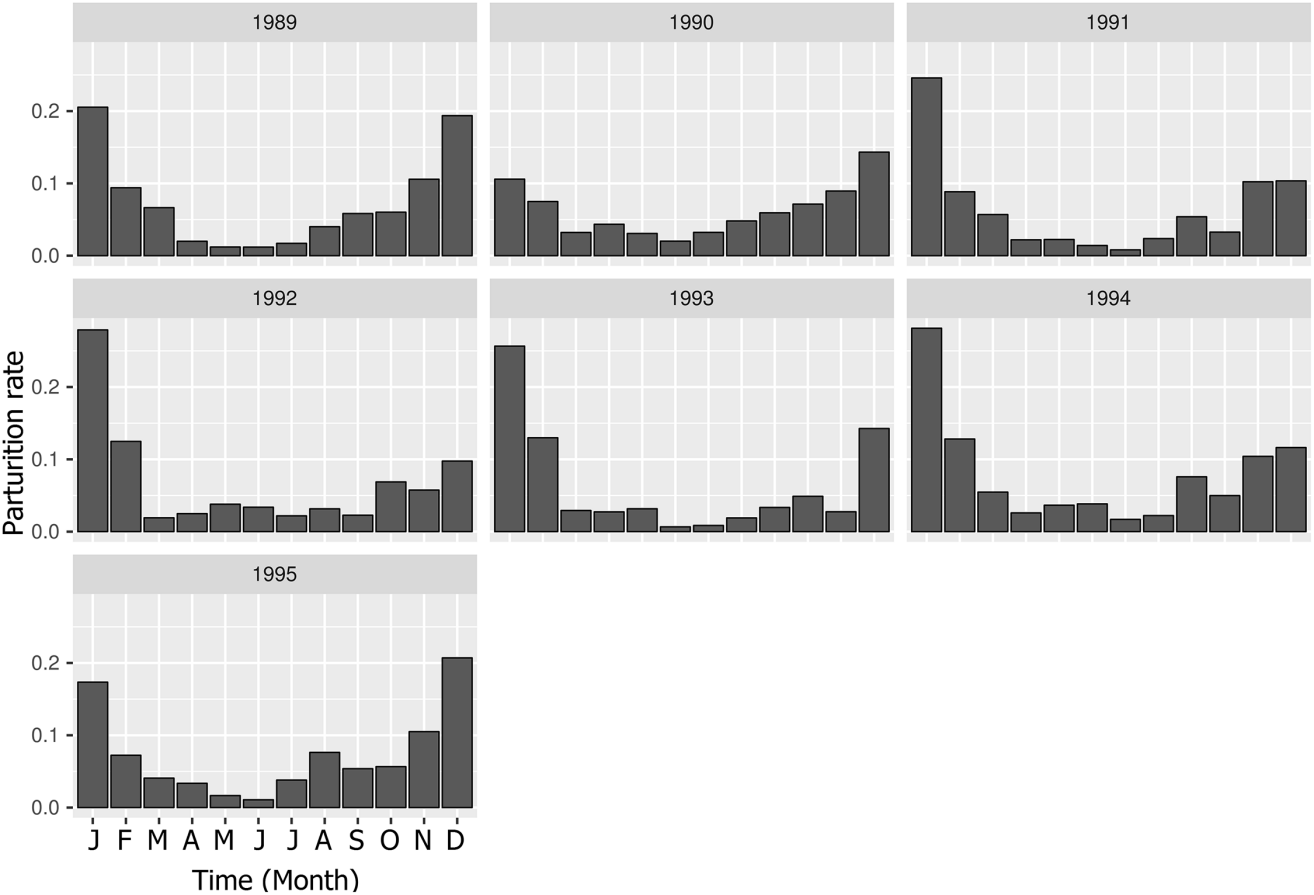
Variations in estimated monthly parturition rates for ewes older than 10 months, Ndiagne municipality, Senegal.

**Fig 4:**
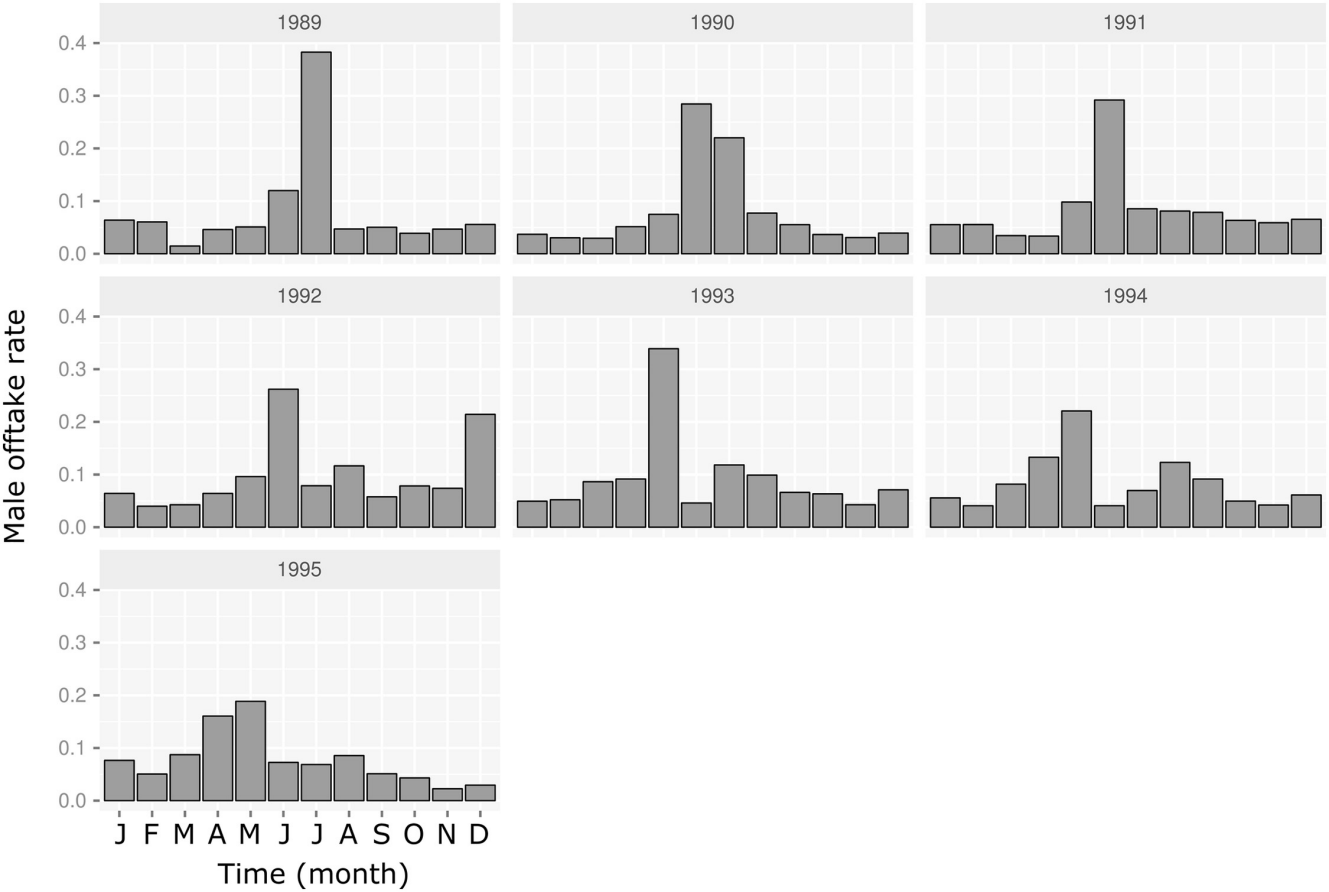
Variations in estimated monthly offtake rates in rams older than 10 months, Ndiagne municipality, Senegal.

As a first step, the statistical models included the year as a dependent variable to compare the estimated and observed seasonal dynamics. When relevant, the other dependent variables were the sex, the age group (juveniles: zero to six months, sub-adults: ≥ six to 10 months, adults: ≤ 10 months) and the season. For all the rates except the male offtake probabilities *p*_*off*_, the year was split into four periods (January to March, April to June, July to September, October to December) to form a “season” dependent variable. The estimation of the male offtake seasonality required a specific approach since one objective of the study was to quantify the effect of the Tabaski month on the *PIR* dynamics according to its month of occurrence. However, in the available data (i.e. between 1989 and 1995), Tabaski only occurred between May and July. To overcome this problem, the seasonal variations of the offtake rate were modeled using a two-level qualitative factor, where the first level was the “Tabaski period” (*T* = months *j* − 1, *j*, *j* + 1 where *j* was the month of Tabaski) and the second level was the “out-of-Tabaski period” corresponding to the nine other months. As an illustration, Figs 3 and 4 show the seasonal demographic patterns occurring in the monitored sheep population.

In a second step, the demographic rates were averaged over the whole study period, by marginalizing the year effect using unweighted means [59], for representing an average year. These averaged rates and their standard errors were used for simulating the *PIR* dynamics and uncertainty. The Tabaski period was successively centred on each month of the year to represent the 12 scenarios of Tabaski month considered in the study. Assuming Gaussian distribution for the average demographic rates estimates [60], 10,000 Monte Carlo replications of the *PIR* dynamics were run with the population matrix model for the 432 scenarios. For each scenario, 95% confidence intervals of the *PIR* outputs were estimated by the 2.5% and 97.5% quantiles of the resulting empirical distributions. For preventing biases in the comparisons of *PIR* dynamics due to artificial demographic effects, the sheep population was assumed, in each replication, to have a constant annual population multiplication rate *m* = 1, where *m* was the ratio between the population size at the end and the start of the year. This was achieved by a simple adjustment of the adult female offtake rates [61, 62] after all the rates were simulated from the Gaussian distributions.

The population matrix model was developed with the mmage add-on package [63] for the R software environment for statistical computing and graphics [64].

## Results

The estimated demographic parameters and associated standard errors are provided as MS Excel files in S2 Table.

### Demographic patterns

**Fig 5:**
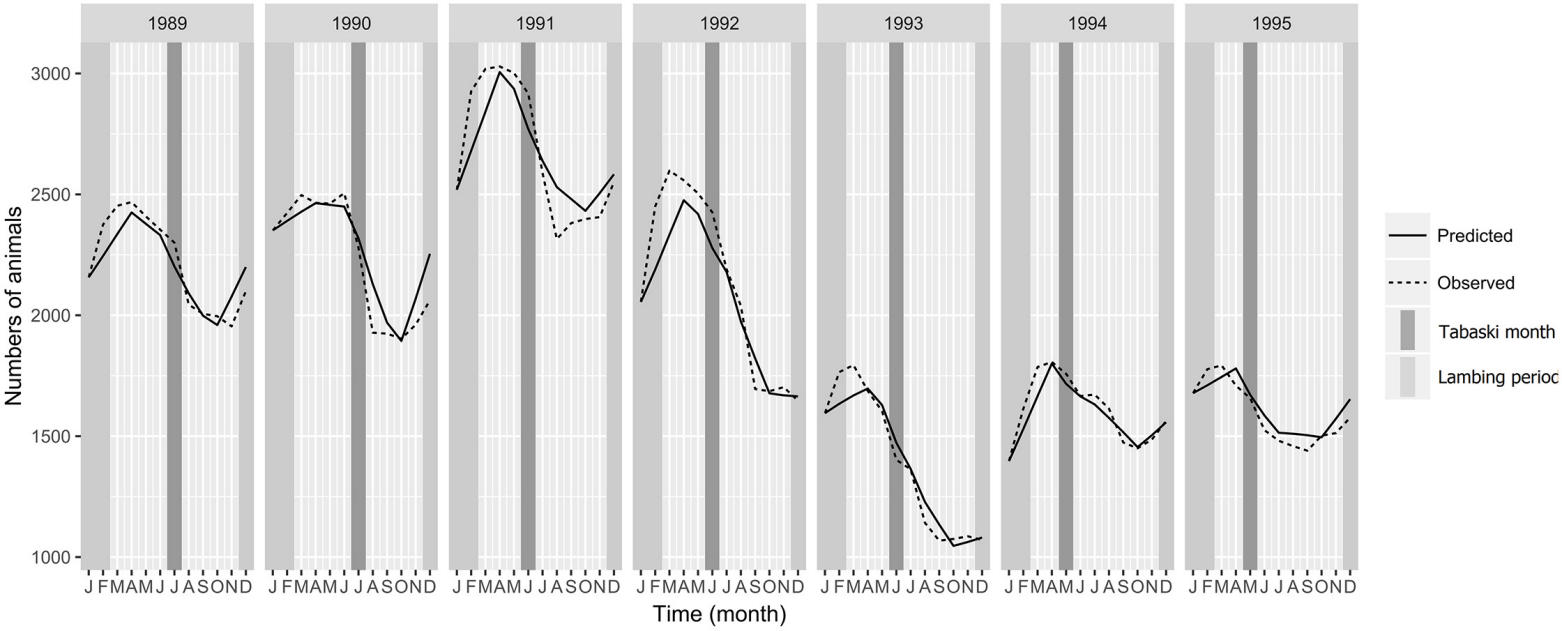
Comparison between simulated (solid line) and observed (dashed line) sheep population dynamics from 1989 to 1995, Ndiagne municipality (Senegal). Lambing period and Tabaski celebration are represented by the vertical light and dark gray strip.

The population dynamics model accurately reproduced the demographic patterns of Ndiagne’s sheep population for the seven simulated years (Fig 5; Spearman’s correlation coefficient between simulated and empirical data was $\hat{\rho }=0.97$ (*P* < 10^−4^). Driven by the parturition peak (Fig 3), the population size increased from October-November to March-April (Fig 5). With the quasi-absence of births during the dry season (Fig 3), it decreased during the rest of the year due to animal exits (natural mortality and offtake) (Fig 5).

**Fig 6:**
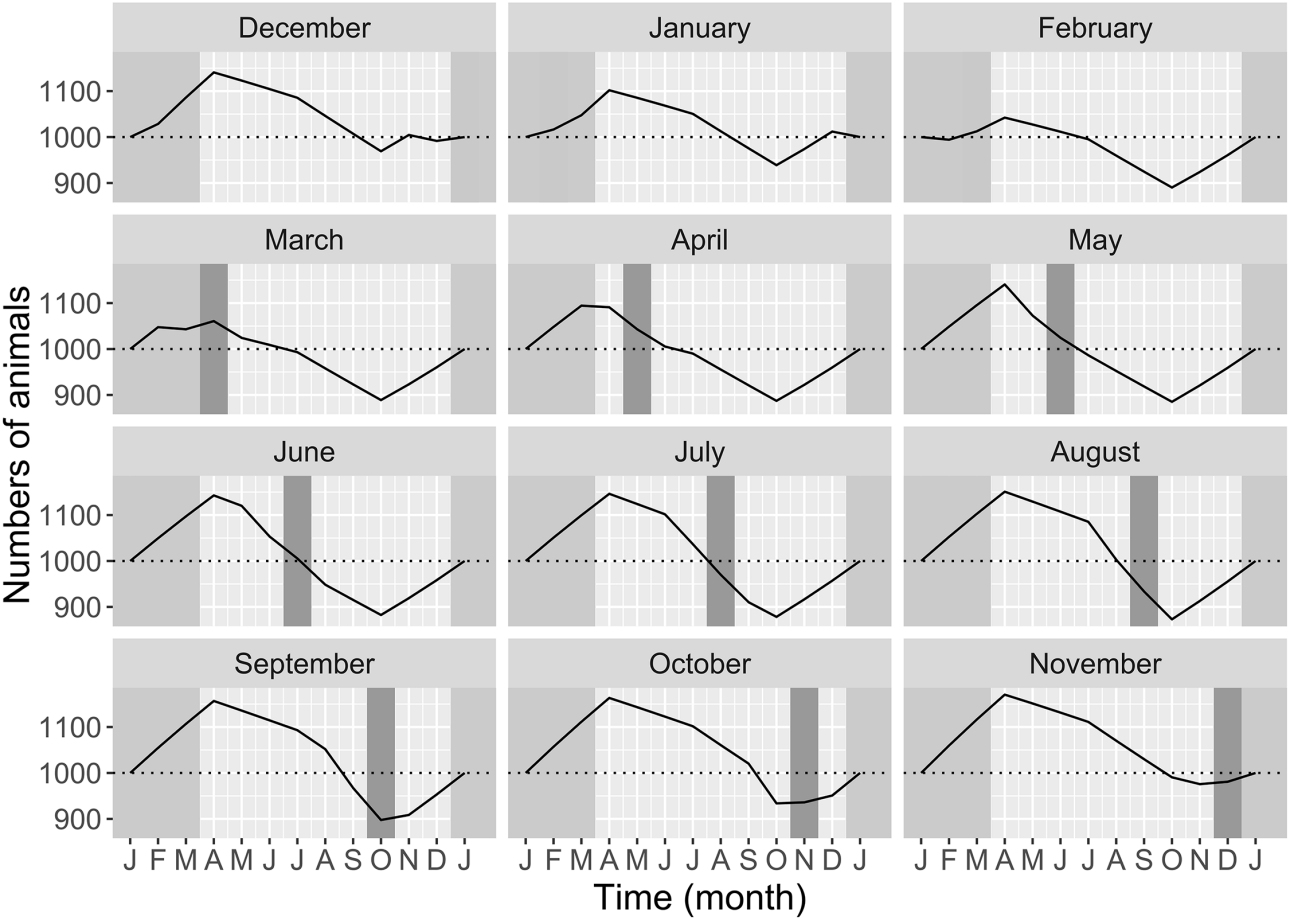
Seasonal population dynamics in Sahelian sheep, Ndiagne municipality, Senegal simulated under 12 Tabaski scenarios. Tabaski month is represented by a dark gray vertical strip and the lambing season by a light gray vertical strip (December to February). a) Tabaski occurring during the lambing season; b) Tabaski occurring soon after the lambing season; c) Tabaski occurring long after the lambing season; d) Tabaski occurring before the lambing season.

**Fig 7:**
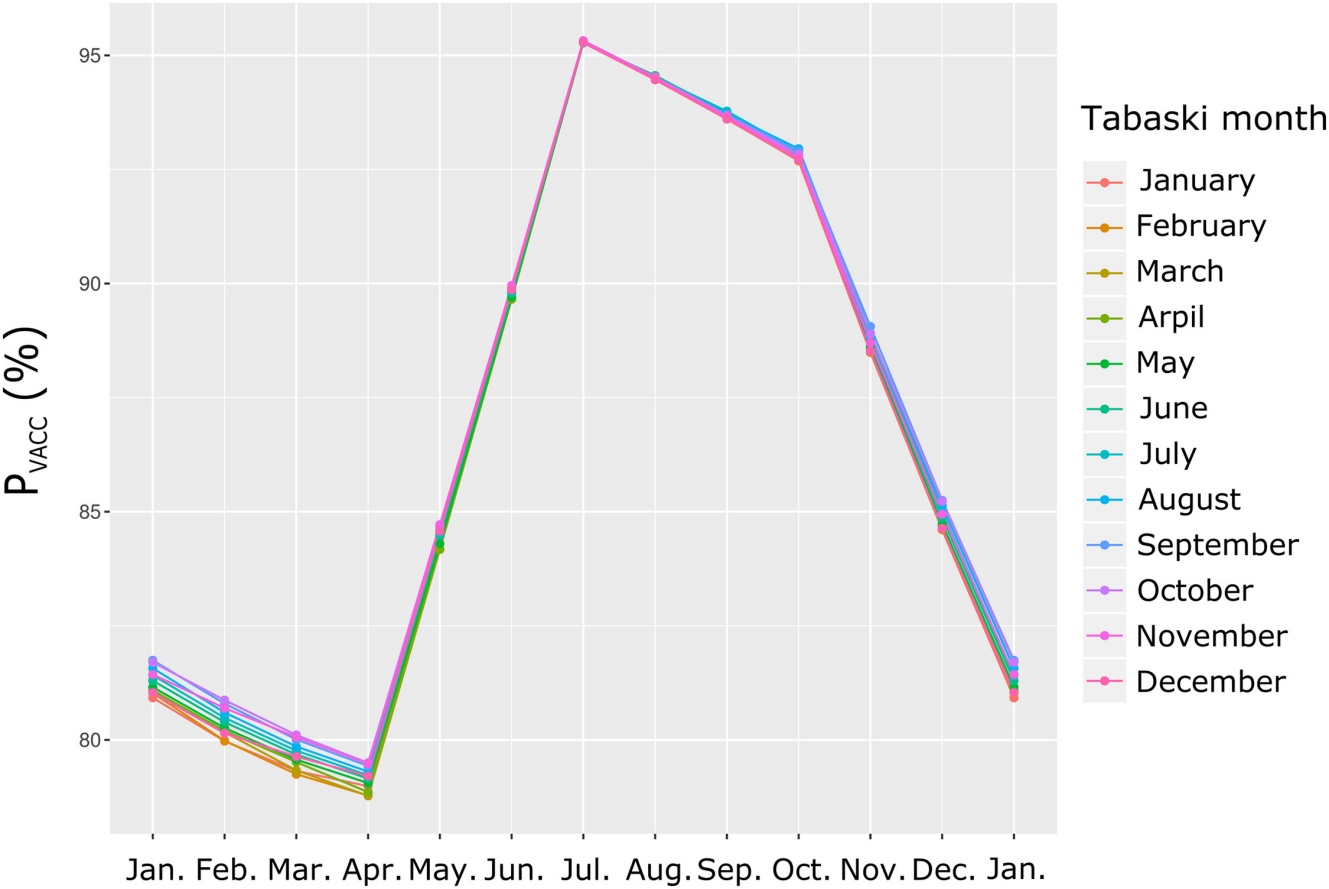
Estimated monthly proportion of animals older than 3 months according to the Tabaski month (*P*_*vacc*_).

The 12 demographic models built with varying Tabaski month showed similar demographic patterns (Fig 6). Nevertheless, the amplitude of demographic variation was different among the Tabaski scenarios. When the Tabaski occurred during the lambing season (December-February), the seasonal variations were limited (Fig 6a). The impact of births on demography was masked by the male offtake. When Tabaski occurred soon after the lambing season, the population peak was higher than in the previous case but was limited to the beginning of the year; the population size quickly decreased thereafter because of both the Tabaski offtake peak and the minimal reproduction (Fig 6b). When the Tabaski occurred long after the lambing season, the seasonal variations were maximal. Offtake related to Tabaski exacerbated the population decay (Fig 6c and 6d). When Tabaski occurred before the lambing period, its effect on population size was minimal (Fig 6d). However, the estimation of *P*_*vacc*_ showed that the proportion of animals belonging to the vaccinated cohort varied over the year, reaching its maximum in July and its minimum in April, whatever the Tabaski month (Fig 7). Therefore, the Tabaski festival did not influenced the population structure between young (≤ 3 months) and juveniles/adults (>3 months).

### *PIR* dynamics

**Fig 8:**
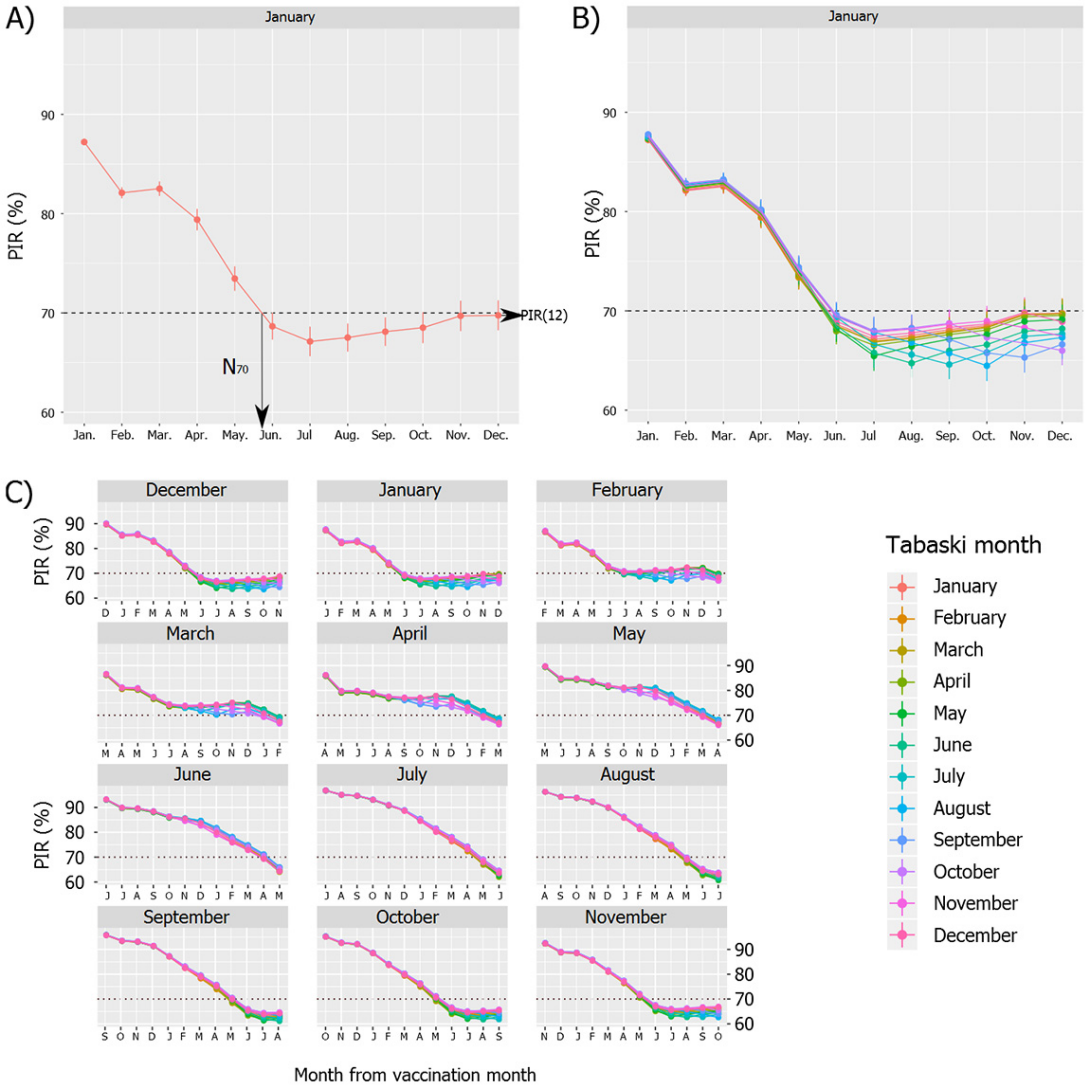
Annual dynamics of post-vaccination *PIR* in Sahelian sheep, Ndiagne municipality (Senegal), assuming full vaccination coverage (*p* = 1). A) Vaccination campaign and Tabaski in January; B) Vaccination campaign in January and changing Tabaski month; C) Changing vaccination month (12 plots) and Tabaski month (12 lines). On each plot, the origin of the *x* axis is the vaccination month.

The *PIR* dynamics obtained assuming full vaccination coverage (*p* = 1) is shown on Fig 8.

Fig 8A shows the result of a vaccination campaign and Tabaski both occurring in January. The starting point (upper left) of the curve was the proportion of immunized sheep at the end of the vaccination campaign. Then, the simulated *PIR* decreased and fell under the threshold of 70% until reaching a plateau. The starting point was 84.2% [86.7; 87.8] (95% confidence interval in brackets). This value depended on the vaccination coverage and the proportion of susceptible animals in the unvaccinated cohort (lambs ≤ three months). Because the vaccination coverage was 100%, the susceptible animals (15.8%) were lambs having lost their colostral immunity. The *PIR* remained higher than 70% during 6.4 months [5; 11] after the vaccination campaign and reached 68.5% [65.7; 71.4] in July.

Fig 8B shows the results of a vaccination campaign in January associated with each of the 12 Tabaski months: the 12 distinct curves were nearly superimposed, showing that for this vaccination month, the *PIR* dynamics did not vary according to the Tabaski month.

Fig 8C shows the results of the 144 scenarios: 12 vaccination months (12 plots) × 12 Tabaski months (12 lines per plot). On the 12 plots, the 12 lines are nearly superimposed, showing that, whatever the vaccination month, the Tabaski month did not affect the *PIR* dynamics. However, substantial differences in *PIR* pattern were observed according to the vaccination month.

**Fig 9:**
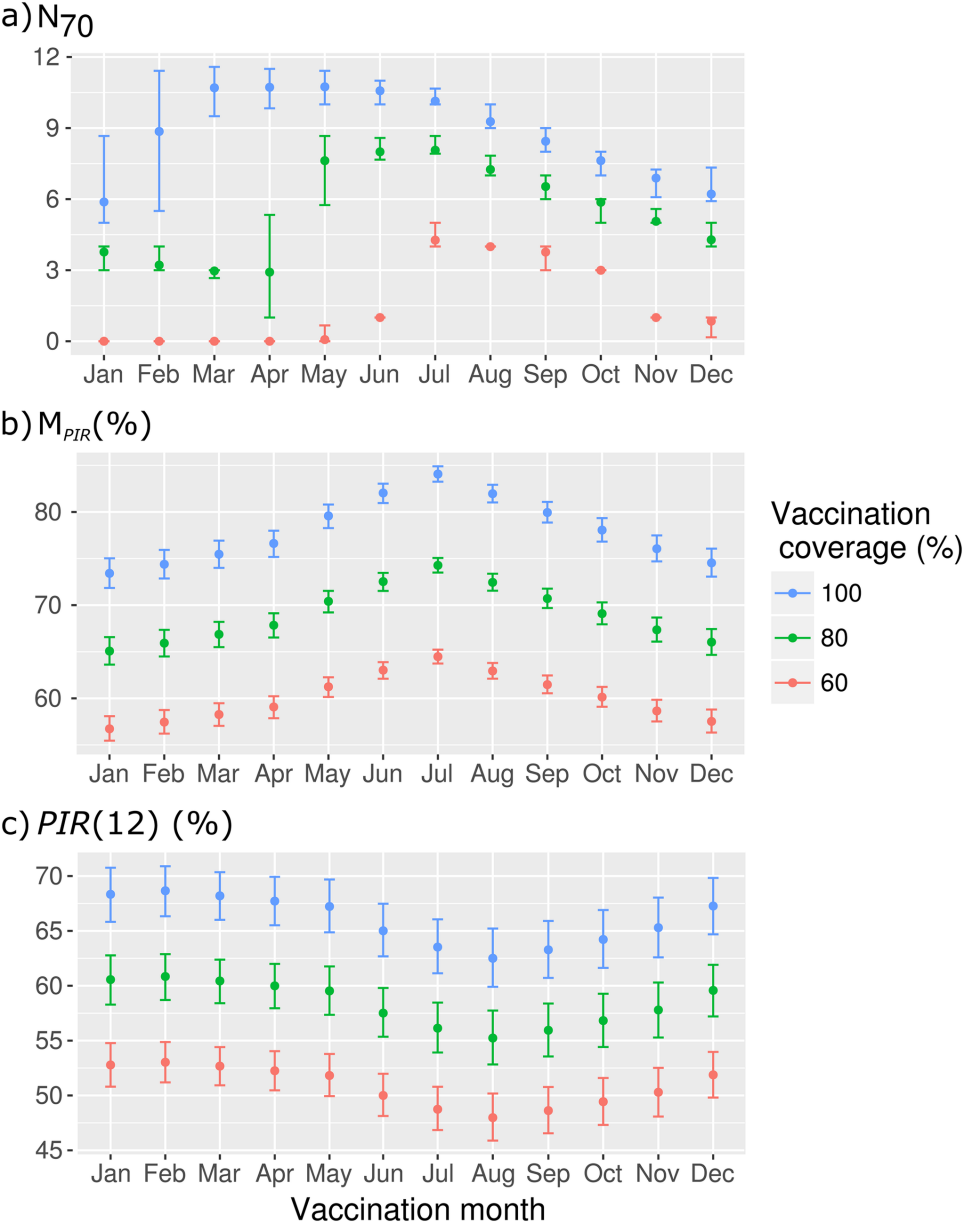
*PIR* indicators according to the simulated vaccination month: *N*_70_, *M*_*PIR*_, and *PIR*(12). From the top to the bottom, the plots show (a) the number of months for which *PIR*(*t*) ≥ 70% (*N*_70_), (b) the mean annual *PIR* (*M*_*PIR*_) and (c) the *PIR* one year after the vaccination campaign (*PIR*(12)).

- When vaccination occurred during the lambing season (December-February first row of plots), the proportion of immunized animals in the population at the end of the vaccination campaign (*PIR*(1)) was 88.1% [86.7; 90.1], i.e. well above the 70% threshold. The *PIR* slowly decreased during a few months, then sharply dropped before reaching a plateau around July: *PIR*(12) = 68.1% [65.4; 70.6], Fig 9B. The number of months with *PIR*(*t*) > 70% was highly sensitive to the demographic parameters: *N*_70_ = 7 [5;11], Fig 9C. The mean population immunity rate over the year (*M*_*PIR*_) was 74.1% [72.4; 75.8], Fig 9.
- When vaccination occurred just after the lambing season (March-May; Fig 8C, second row of plots), the *PIR*(1) were similar (87.3%, [85.8; 89.7]) because many lambs were older than three months at the vaccination date. The *PIR* decay was slow and reached *PIR*(12) = 67.7% [65.3; 70.1] at the end of the year. It remained above 70% during *N*_70_ = 11 months [10; 12]. The *M*_*PIR*_ were 77.2% [74.5; 80.4] (Fig 9).
- When vaccination occurred long after the lambing season (June-August; Fig 8C, third row of plots), the *PIR*(1) were higher (95.4% [93.1; 96.9]) because most lambs were old enough to be vaccinated at the vaccination date. After a short plateau, the *PIR* decreased and reached 70% after *N*_70_ = 10 [9; 11] months. The *PIR*(12) were 63.7% [60.7; 66.7]. *M*_*PIR*_ were 82.7% [81.1; 84.6] (Fig 9).
- When vaccination occurred within three months before the lambing period (September-November; Fig 8C, fourth row of plots), the *PIR*(1) were still high (94.5% [92.3; 95.8]) because few offspring were born during the three previous months. The *PIR* dynamics followed a plateau till the lambing season, then quickly decreased to reach a second plateau in June / July with *PIR*(12) = 64.3% [61.3; 67.3]. The *PIR* remained above 70% over *N*_70_ = 8 [7; 9] months with *M*_*PIR*_ = 78.0% [75.2; 80.4] (Fig 9).

With vaccination coverage set to *p* = 0.8, *PIR* patterns were similar to those with full vaccination coverage (see Supporting information S1 Fig). The global indicators were drawn in green, Fig 9. The range of *PIR* dropped by 7–11% (Fig 9b and 9c). Nevertheless, the vaccination scenarios providing the highest *N*_70_ were obtained for vaccination campaign implemented between May and November. With vaccination campaigns between January and April, *N*_70_ was lower than four months (Fig 9a).

With vaccination coverage set to *p* = 0.6, *PIR* patterns were similar to those with higher *p* (S2 Fig). The global indicators are shown in red in Fig 9. The range of values dropped again by 7–11%. The *PIR* briefly reached the 70% threshold when vaccination campaigns were implemented between July and October (*N*_70_ = [1; 5], Fig 9a), because of the presence of animals initially immunized, including colostral immunity in offspring. Otherwise, when vaccination was simulated between December and May, *N*_70_ = 0 or 1, the single favorable month being the vaccination month.

## Discussion

### Tabaski month

Simulations showed that, for PPR vaccination in an epidemiological unit taken from a Sahelian smallholder sheep farming system, the *PIR* dynamics were mostly influenced by the date of the vaccination campaign, and much less by the date of the Tabaski festival. The most prominent Tabaski effect on demographic parameters was a strong, seasonal increase of male offtake in age classes around 1 year old. The Tabaski strongly influenced the shape of demographic patterns, but not the population structure when considering 2 age classes: younger or older than 4 months. The effect of Tabaski on *PIR* dynamics was probably low because, in these farming systems, most adult sheep are ewes (irrespective of Tabaski date). As a matter of fact, farmers utilize the young rams which they consider as the economic interest of their productive capital (sheep herd), to cover their familial needs. For sheep herds of regular size in this farming system (say 20–50 adult ewes), they only keep one adult ram for mating purpose [25, 26]. For those farmers, Tabaski festival is an opportunity to sell young rams which would have been utilized anyway. In addition Tabaski-related offtake do not involve the adult females who provide colostral antibodies to their offspring, nor the young rams from unvaccinated age classes. Therefore, the proportion of vaccinated sheep during the campaign is only weakly related to the Tabaski date.

### Vaccination month

The study of global indicators showed that the *PIR* was never kept above the 70% threshold over the whole year: *PIR*(12) < 70%, and *N*_70_ < 12 (Fig 9B). The residual post-vaccination immunity rate one year after vaccination was little influenced by the vaccination date. Nonetheless, the highest rates were obtained when the vaccination campaign occurred between December and May: in these conditions, the lambing peak occurred one year after vaccination, thus resulting in a high number of immunized lambs (colostral antibodies) in the sheep population.

Simulations showed that PPR vaccination campaigns implemented from April to July would provide the highest post-vaccination immunity rate. Indeed, such vaccination campaigns would theoretically cover a larger part of the population (highest *P*_*vacc*_) than during other periods, because of the quasi-absence of lambs younger than 3 months. Moreover, because of the low number of lambing till December, the *PIR* remained at a high level during a long time.

### Vaccination coverage

The reduction of vaccination coverage from 100% to 80% (-20%) did not proportionally reduce the *PIR*(12) (-7 to -11%). As a matter of fact, the existence immunized females in the population (before vaccination) “buffers” the reduction of vaccination coverage because they provide colostral antibodies to their offspring, irrespective of their vaccination status. This has important practical consequences, because trying to reach 100% vaccination coverage is difficult and expensive in most cases: given the lack of vaccination facilities in most cases (pens, corridors, etc.), sorting and catching all the sheep is cumbersome. On the other hand, convincing a majority of farmers to bring a large proportion of their animals for vaccination is not an impossible task. For instance, after many years of mass vaccination against rinderpest in Senegal, the population immunity rate reached 70% in the general cattle population [65]. Similar figures were reported after the mass vaccination campaigns implemented during three successive years in Morocco following PPR emergence in 2008. Though PPR was eliminated from this country during several years, with no evidence of PPRV circulation according to a strict surveillance program, the post-vaccination immunity rate was only 70% in the general sheep population [33]. Of course, the major problem in such situations would be to make sure that no sub-population pocket exists where the proportion of PPRV-susceptible animals remains high, thus making possible the virus persistence in the environment. Indeed, simulation studies are not enough: carefully designed field epidemiological studies are undoubtedly needed to assess the actual post-vaccination immunity level.

Moreover, when we assessed imperfect vaccination coverage (*p* = 80% or 60%), *PIR*(12) reached 48% or 41%, whatever the vaccination month. Though these rates might be insufficient to fully stop PPRV transmission, they would represent a noticeable achievement for the first year of vaccination. Indeed, because the Global strategy relies on two mass vaccination campaigns targeting all the eligible sheep, the initial coverage from the second campaign would benefit from the former *PIR*(12). Implementing two successive vaccination campaigns might provide more optimistic results.

The main difference related to the vaccination coverage was observed for the *N*_70_. With lower vaccination coverage, the vaccination campaigns implemented at the beginning of the year (January to May) were not able to cover the sheep population with high immunity rates. Under our assumptions, only vaccination campaigns achieved between June and November allowed reaching the 70% threshold for more than one month (*N*_70_ > 1). According to our modeling results, we would recommend to implement vaccination during this period, with a slight preference for scenarios with vaccination campaigns occurring between July and August.

### Comparison with OIE & FAO recommendations

Nevertheless, PPR mass vaccination campaigns cannot be decided on the single basis of simulated immunity rates. As a matter of fact, vaccination campaigns are unwelcome during the rainy season (from July to September) because farmers are busy with the rain-fed crops: groundnuts, millet, etc. [66–68]. Also, village accessibility is often an issue for vaccination teams because of flooding and impracticable roads and tracks.

The implementation of vaccination campaigns is not suitable during the warm, dry season (April to June), commonly called the “hunger gap”. At this period, farmers’ cash reserves are at their lowest level: there are no more income from crops. Therefore, they cannot afford for the sheep vaccination fees. Moreover, forage availability is at its minimum level: most sheep are underfed and in a poor body condition. In this physiological status, the immunized system of sheep does not operate correctly, with a low globulin rate and a reduced immunized response to the vaccine [69–72]. In addition, sheep are left straying freely and they need to be gathered for the vaccination, which is a cumbersome work for the farmers at this period.

As a consequence of these constraints, a trade-off has to be found between suggestions derived from our simulation studies and practical considerations. Following our results, a possible recommendation would be to implement the vaccination campaign as soon as possible after July to reach appropriate *PIR* and keep it at a high level. On the other hand, given the above-mentioned constraints, the officially recommended period for the implementation of PPR vaccination campaigns in arid and semi-arid regions such as in the Ndiagne area, is between September and November, i.e. at the end of the rainy season and before the lambing season (Appendix 3.4 in [7]). Though this time period did not provide the best simulated indicators, it is still acceptable in terms of *PIR*.

Planning disease control requires knowledge and tools to adapt the strategy and maximize the efficiency of control actions. In the best cases, the strategies are based on the results of disease surveillance and other epidemiological studies [73, 74], disease transmission models [21, 75], and economic studies [2, 76, 77]. Post-vaccination evaluation of vaccination campaigns use similar tools and methods [7, 78, 79]. In this frame, the use of models is relatively new [17, 80]. We believe that the improvement of the method initially developed by Lesnoff *et al*. [17] provides an useful tool for *ex ante* assessment of vaccination strategies. It might be adapted to other vaccination programs for any other domestic species as long as as accurate and long-term demographic data are available.

Further work might couple this *PIR* model with a virus transmission model such as an SIR model to assess the impact of *PIR* dynamics on PPRV transmission. Our method might also be useful to economists as a corner stone to assess cost / efficiency or cost / benefit ratios for vaccination campaigns.

## Supporting Information

S1 Matrix Population state vector structure and projection matrix. *x*: number of animal for a given sex and age class; *ρ*: proportion of female at birth; *s*: survival rates for a given sex and age class (assuming equal rates for male and female newborn); *f*: reproduction rate for the female belonging to a given age class.
(PDF)

S1 Table Observed demographic data collected in Ndiagne sheep herds (northern Senegal) from 1989 to 1995. The structure of the table is described in the chapter 7.3 of Lesnoff *et al*, 2011 [81] and in the French version freely available at http://livtools.cirad.fr/laserdemog, together with toy datasets and worked examples of analyses.
(CSV)

S2 Table Marginal means and standard errors of monthly demographic parameters. The parameters were estimated for three age classes: juveniles sub-adults and adults (1: zero to six months, 2: ≥ six to 10 months, 3: ≤ 10 months), two sexes (F: female; M: male) and according to four periods (1: January-February-March; 2: April-May-June; 3: July-August-September; 4: October-November-December), except for the male offtake rates which were estimated according only two periods: i) Tabaski: the month preceding the Tabaski, the Tabaski month, the month following the Tabaski and ii) the 9 other months).
(ZIP)

S1 Fig Annual dynamics of post-vaccination *PIR* in Sahelian sheep, Ndiagne municipality (Senegal), assuming 80% of vaccination coverage (*p* = 0.8). A total of 144 vaccination scenarios are represented crossing the vaccination month (12 plots) with the Tabaski month (12 lines). On each plot, the origin of the *x* axis is the vaccination month.
(TIF)

S2 Fig Annual dynamics of post-vaccination *PIR* in Sahelian sheep, Ndiagne municipality (Senegal), assuming 40% of vaccination failure (*p* = 0.6). A total of 144 vaccination scenarios are represented crossing the vaccination month (12 plots) with the Tabaski month (12 lines). On each plot, the origin of the *x* axis is the vaccination month.
(TIF)
