# Supplementary material for: Modelling the Dynamics of Post-Vaccination Immunity Rate in a Population of Sahelian Sheep after a Vaccination Campaign against Peste des Petits Ruminants Virus
Source: PLoS One. 2016 Sep 7;11(9):e0161769. doi: 10.1371/journal.pone.0161769 (PMC5014330; doi:10.1371/journal.pone.0161769)
Supplement: S1 Matrix — x: number of animal for a given sex and age class; ρ: proportion of female at birth; s: survival rates for a given sex and age class (assuming equal rates for male and female newborn); f: reproduction rate for the female belonging to a given age class. (PDF) [file pone.0161769.s001.pdf]

## S1 Matrix

**Population state vector structure and projection matrix.**

$x$: number of animal for a given sex and age class ; $\rho$: proportion of female at birth ; $s$: survival rates for a given sex and age class (assuming equal rates for male and female newborn) ; $f$: reproduction rate for the female belonging to a given age class.

$$
\begin{bmatrix} x_{f,1} \\ ... \\ x_{f,132} \\ \hline x_{m,1} \\ ... \\ x_{m,24} \end{bmatrix}_{t+1}
=
\left[\begin{array}{cccccccc|ccccc}
0 & 0 & ... & 0 & (\rho \times s_0)(f_{11} \times s_{f,11}) & ... & 0 & (\rho \times s_0)(f_{132} \times s_{f,132}) & 0 & 0 & ... & 0 & 0 \\
s_{f,1} & 0 & ... & 0 & 0 & 0 & 0 & 0 & 0 & 0 & ... & 0 & 0 \\
... & ... & ... & ... & ... & ... & ... & ... & ... & ... & ... & ... & ... \\
0 & 0 & ... & 0 & 0 & ... & 0 & 0 & 0 & 0 & ... & 0 & 0 \\
0 & 0 & ... & s_{f,10} & 0 & ... & 0 & 0 & 0 & 0 & ... & 0 & 0 \\
0 & 0 & ... & ... & ... & ... & ... & ... & ... & ... & ... & ... & ... \\
0 & 0 & ... & 0 & 0 & ... & 0 & 0 & 0 & 0 & ... & 0 & 0 \\
0 & 0 & ... & 0 & 0 & ... & s_{f,131} & s_{f,132} = 0 & 0 & 0 & ... & 0 & 0 \\
\hline
0 & 0 & ... & 0 & ((1-\rho) \times s_0)(f_{11} \times s_{f,11}) & ... & 0 & ((1-\rho) \times s_0)(f_{132} \times s_{f,132}) & 0 & 0 & ... & 0 & 0 \\
0 & 0 & ... & 0 & 0 & 0 & 0 & 0 & s_{m,1} & 0 & ... & 0 & 0 \\
... & ... & ... & ... & ... & ... & ... & 0 & ... & ... & ... & ... & ... \\
0 & 0 & ... & 0 & 0 & ... & 0 & 0 & 0 & 0 & ... & 0 & 0 \\
0 & 0 & ... & 0 & 0 & ... & 0 & 0 & 0 & 0 & ... & s_{m,23} & s_{m,24} = 0
\end{array}\right]_{t}
\times
\begin{bmatrix} x_{f,1} \\ x_{f,2} \\ ... \\ x_{f,10} \\ x_{f,11} \\ ... \\ x_{f,131} \\ x_{f,132} \\ \hline x_{m,1} \\ x_{m,2} \\ ... \\ x_{m,23} \\ x_{m,24} \end{bmatrix}_{t}
$$
