# Supplementary figures and images for: Modelling the Dynamics of Post-Vaccination Immunity Rate in a Population of Sahelian Sheep after a Vaccination Campaign against Peste des Petits Ruminants Virus
Source: PLoS One. 2016 Sep 7;11(9):e0161769. doi: 10.1371/journal.pone.0161769 (PMC5014330; doi:10.1371/journal.pone.0161769)

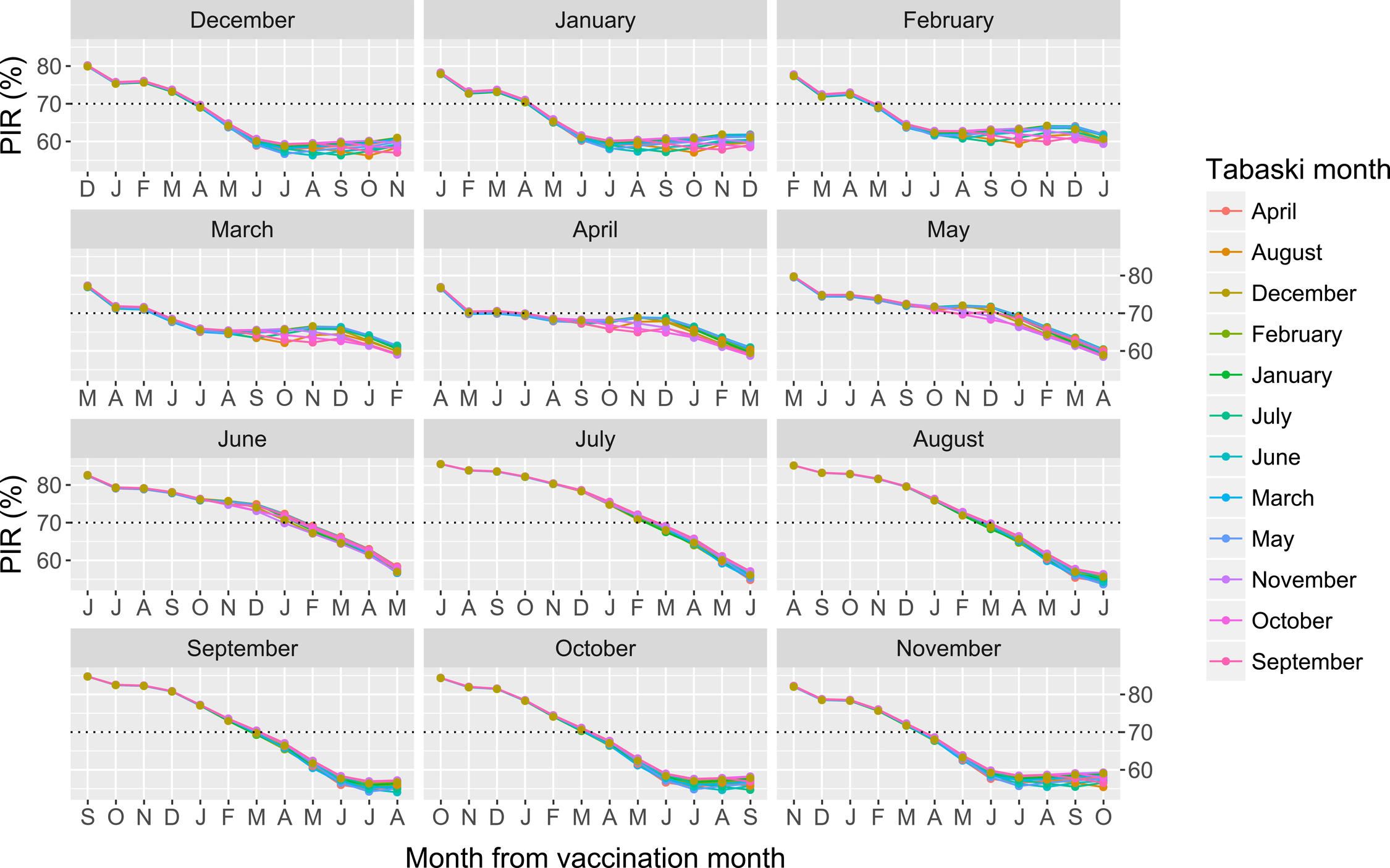

Supplement: S1 Fig — A total of 144 vaccination scenarios are represented crossing the vaccination month (12 plots) with the Tabaski month (12 lines). On each plot, the origin of the x axis is the vaccination month. (TIF) [file pone.0161769.s004.tif]

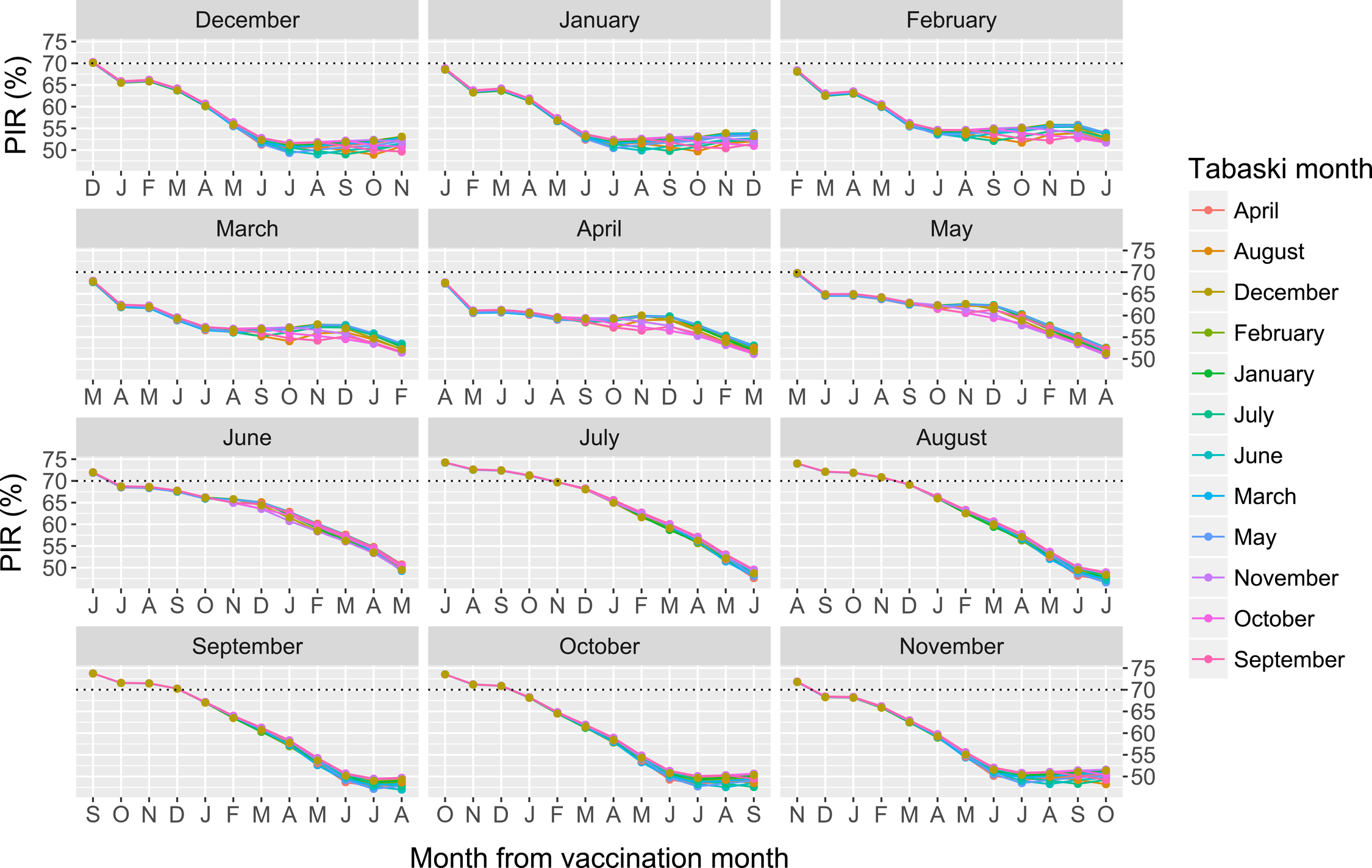

Supplement: S2 Fig — A total of 144 vaccination scenarios are represented crossing the vaccination month (12 plots) with the Tabaski month (12 lines). On each plot, the origin of the x axis is the vaccination month. (TIF) [file pone.0161769.s005.tif]
